# A Participatory System for Preventing Pandemics of Animal Origins: Pilot Study of the Participatory One Health Disease Detection (PODD) System

**DOI:** 10.2196/publichealth.7375

**Published:** 2018-03-21

**Authors:** Terdsak Yano, Somphorn Phornwisetsirikun, Patipat Susumpow, Surasing Visrutaratna, Karoon Chanachai, Polawat Phetra, Warangkhana Chaisowwong, Pairat Trakarnsirinont, Phonpat Hemwan, Boontuan Kaewpinta, Charuk Singhapreecha, Khwanchai Kreausukon, Arisara Charoenpanyanet, Chongchit Sripun Robert, Lamar Robert, Pranee Rodtian, Suteerat Mahasing, Ekkachai Laiya, Sakulrat Pattamakaew, Taweesart Tankitiyanon, Chalutwan Sansamur, Lertrak Srikitjakarn

**Affiliations:** ^1^ Faculty of Veterinary Medicine Chiang Mai University Chiang Mai Thailand; ^2^ Department of Livestock Development Bangkok Thailand; ^3^ Opendream Co Ltd Bangkok Thailand; ^4^ Chiang Mai Provincial Public Health Office Chiang Mai Thailand; ^5^ Faculty of Political Science Chiang Mai University Chiang Mai Thailand; ^6^ Faculty of Social Sciences Chiang Mai University Chiang Mai Thailand; ^7^ Faculty of Medicine Chiang Mai University Chiang Mai Thailand; ^8^ Faculty of Economics Chiang Mai University Chiang Mai Thailand

**Keywords:** community-owned disease surveillance system, PODD, mobile app, one health, participatory approach, backyard chicken, pandemic prevention

## Abstract

**Background:**

Aiming for early disease detection and prompt outbreak control, digital technology with a participatory One Health approach was used to create a novel disease surveillance system called Participatory One Health Disease Detection (PODD). PODD is a community-owned surveillance system that collects data from volunteer reporters; identifies disease outbreak automatically; and notifies the local governments (LGs), surrounding villages, and relevant authorities. This system provides a direct and immediate benefit to the communities by empowering them to protect themselves.

**Objective:**

The objective of this study was to determine the effectiveness of the PODD system for the rapid detection and control of disease outbreaks.

**Methods:**

The system was piloted in 74 LGs in Chiang Mai, Thailand, with the participation of 296 volunteer reporters. The volunteers and LGs were key participants in the piloting of the PODD system. Volunteers monitored animal and human diseases, as well as environmental problems, in their communities and reported these events via the PODD mobile phone app. LGs were responsible for outbreak control and provided support to the volunteers. Outcome mapping was used to evaluate the performance of the LGs and volunteers.

**Results:**

LGs were categorized into one of the 3 groups based on performance: A (good), B (fair), and C (poor), with the majority (46%,34/74) categorized into group B. Volunteers were similarly categorized into 4 performance groups (A-D), again with group A showing the best performance, with the majority categorized into groups B and C. After 16 months of implementation, 1029 abnormal events had been reported and confirmed to be true reports. The majority of abnormal reports were sick or dead animals (404/1029, 39.26%), followed by zoonoses and other human diseases (129/1029, 12.54%). Many potentially devastating animal disease outbreaks were detected and successfully controlled, including 26 chicken high mortality outbreaks, 4 cattle disease outbreaks, 3 pig disease outbreaks, and 3 fish disease outbreaks. In all cases, the communities and animal authorities cooperated to apply community contingency plans to control these outbreaks, and community volunteers continued to monitor the abnormal events for 3 weeks after each outbreak was controlled.

**Conclusions:**

By design, PODD initially targeted only animal diseases that potentially could emerge into human pandemics (eg, avian influenza) and then, in response to community needs, expanded to cover human health and environmental health issues.

## Introduction

### Pandemics

As an estimated 61% of human pathogens are zoonoses [1], a key strategy to avert pandemics is the early detection of pathogen occurrence or disease outbreak in domestic animals and management of the outbreak, so that disease transmission from animal to human populations can be prevented. This is particularly true of influenza pandemics, which can be triggered by emerging avian influenza (AI) viruses. In 2004, an AI outbreak (subtype H5N1) resulted in more than 500 human infections worldwide. On January 23, 2004, in Thailand, an AI outbreak (subtype H5N1) was confirmed in a layer chicken farm by the National Library of Thailand [2]. An abnormal death in a backyard chicken, which is an early indicator of an AI outbreak, is especially worrisome in countries where animal health services need to be improved. The link between disease in backyard chickens and the potential for a global human influenza pandemic requires a holistic view of human, animal, and environmental health. Community participation and a One Health approach—enabling early reporting of suspected cases and implementing effective control measures—played a key role in controlling and eradicating the 2004 H5N1 outbreak in Thailand [3].

### Early Detection

Recognizing the key roles that community participation and a One Health approach play in averting AI pandemics, coupled with the US Flu Near You system having previously demonstrated how participatory reporting using digital tools can help detect influenza outbreaks in human populations faster than traditional surveillance [4], we developed a new type of surveillance tool to enable early detection of potential zoonotic events using a participatory, digital approach grounded in One Health concepts. The system was developed in Chiang Mai, Thailand, and is called Participatory One Health Disease Detection (PODD). This paper describes the following: (1) the core functions of PODD; (2) PODD system’s emphasis on participatory surveillance and community empowerment; (3) PODD system’s initial focus on animal health; (4) results of a performance evaluation of local governments (LGs) and volunteer reporters participating in PODD; and (5) initial effectiveness of PODD in preventing potential AI outbreaks.

## Methods

### Participatory One Health Disease Detection Surveillance Core Functions: Data Reporting, Analysis, and Response

The PODD system is a digital disease surveillance tool with three parts: (1) data reporting to a data collection system via a mobile phone; (2) automated outbreak capturing (ie, automated matching of each reported case or event with case definitions); and (3) automated notification of suspected outbreaks to local governments (LGs), surrounding villages, and all relevant authorities so that they can closely monitor the situation and immediately implement the LG contingency plan to stop the spread of disease.

Volunteer reporters played an important role in reporting. Each LG selected 4 people, who live in the community, who have the willingness to be a volunteer, and who have experience about livestock raising, to be PODD volunteers in the community. A total of 300 volunteers from 75 LGs were recruited in the project. After being trained about how to use PODD and important clinical signs in animals and humans, volunteer reporters continue to meet their trainers every 3 months to be updated on new content and new features of the app and to have their reporting skills tested and improved.

**Figure 1:**
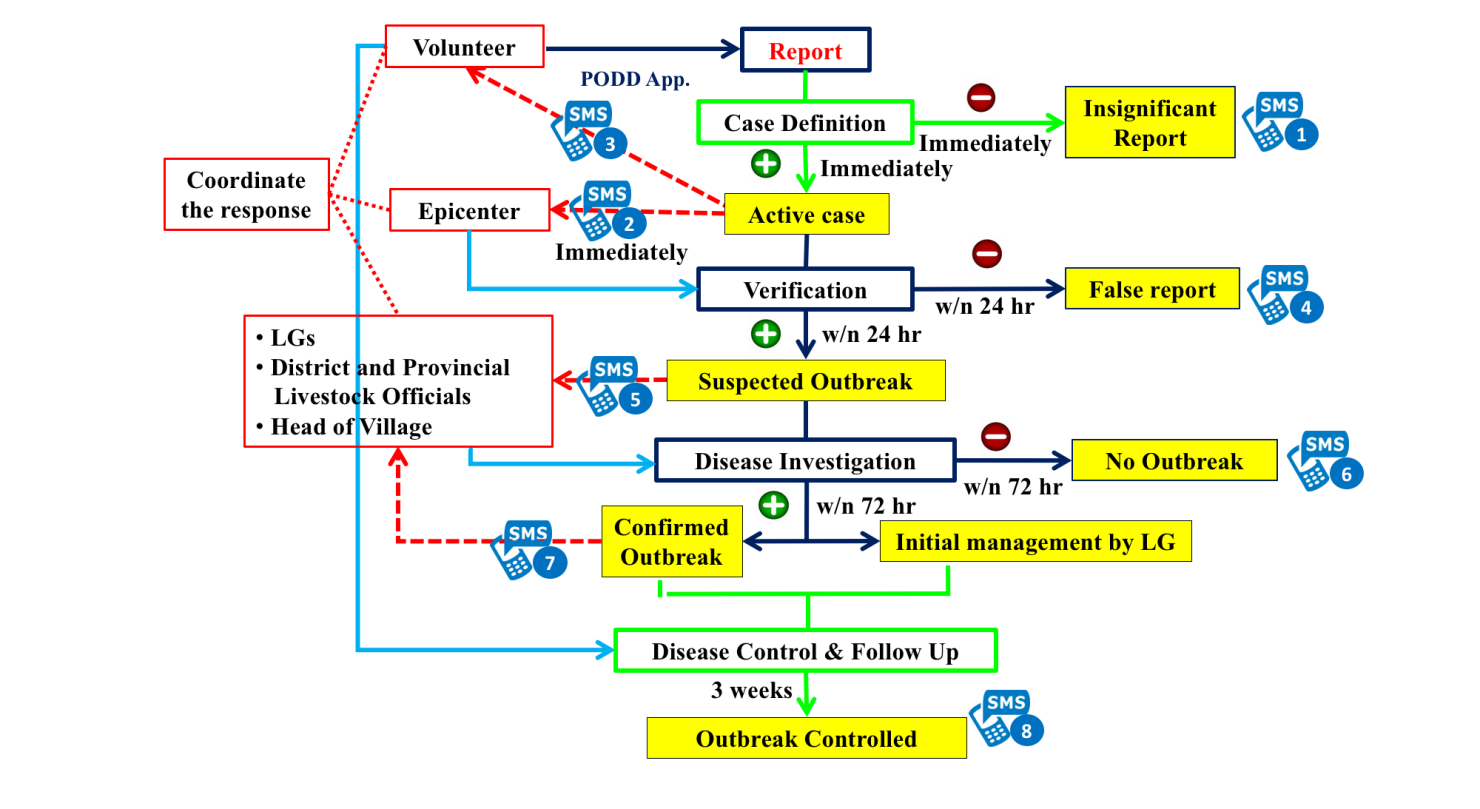
Detailed Participatory One Health Disease Detection (PODD) system workflow indicating roles and responsibilities to control animal diseases. LG: local government; SMS: short message service.

Volunteers reported abnormal animal sicknesses and deaths, animal diseases, animal bites, food safety issues, human diseases, and environmental problems. For abnormal events in animal or animal diseases, the volunteers reported via a PODD mobile app in the animal section. As shown in Figure 1, an automated system matched each report with potential case definitions immediately and assigned a “case” status. In case of a mismatch, the status of the reports was immediately changed to “insignificant report.” If there was a match, reported data were immediately sent to the PODD epicenter, where the epicenter staff, who were mostly veterinarians, verified the reported data by calling reporters and confirming the details within 24 hours. The status of any given report was changed to “suspected outbreak” only after the report had been verified. Upon verification, a short message service text message and an email alert was automatically sent to all stakeholders (ie, researchers, PODD staff, officers from provincial and district Department of Livestock Development [DLD], and LG staff). Additionally, the reporter was given a set of guidelines to manage and to contain the outbreak promptly; LGs and local authorities were notified to activate the LG contingency plan; and district and provincial DLD and public health officers were sent warning messages about the suspected case. Meanwhile, epicenter staff and the district livestock officer, together with the volunteer reporter and other community volunteers, continued to investigate and collect field samples for laboratory confirmation. If the investigative team did not detect an epidemic disease and decided that the case was not a serious issue, the “suspected outbreak” status was changed to “finish.” Despite this, the volunteer reporter was asked to continue the monitoring of the case once a week. If the investigative team detected multiple cases of an infectious disease in either a household or a village, the “suspected outbreak” status was changed to “outbreak.” At this stage, all stakeholders were requested to follow their LG’s contingency plan to control the disease. The “outbreak” status was changed to “finish” only after the outbreak had been controlled.

For human health and environmental problems, volunteers could report via the human and environmental sections, separately. The information of an individual report was sent to PODD system’s data center. Staff at the Provincial Public Health office reviewed the information and allocated it to the specific section to verify and respond to the report. The epidemiology section was responsible for human diseases and zoonosis, the food safety section was responsible for food safety issues, and the environmental section was responsible for environmental problems and public nuisance. The verification process in human health and environmental problems involved calling the reporter, who reported the event, and examining the photograph that the reporter sent via the app.

All events—related to animal health, human health, or environmental problems—were sent to the PODD epicenter to detect whether any relationship exists among them with respect to place or time. The analysis was performed weekly by the staff and reported monthly to researchers. If the link between different events was detected, animal and public health authorities and communities would be notified. Then, disease investigation and response would be processed collaboratively by the animal health authority, the public health authority, and the LG.

In Chiang Mai, the pilot area, the Provincial One Health Committee had been set up, for more than 10 years, to prevent and control issues related to animal health, human health, and environmental problems. This organization had taken the responsibility of serious cases, which were notified from the epicenter and the LG staff in the affected community.

### Participatory One Health Disease Detection System’s Participatory Approach and Community Empowerment

PODD was designed as a community-owned surveillance system that focuses on immediate community benefits, rather than the longer-term goals typically associated with traditional surveillance systems. As described previously and in Figure 1, the PODD system was designed for villagers to serve as volunteer reporters and LGs to take responsibility for controlling outbreaks within their jurisdictions. For piloting the PODD system, 3 LGs in each of the 25 districts in Chiang Mai Province were involved in the project. Due to logistical difficulties, one LG was forced to exit the pilot program. Therefore, 74 LGs covering 296 villages in all regions of Chiang Mai Province participated in the pilot study. The pilot study area covered urban as well as rural areas, including mountainous area. The PODD research team convened the volunteers and LGs every 2 to 4 months to assess progress and discuss potential changes to the system. Critical activities were designed together before implementation.

As community empowerment was the primary goal of PODD, the PODD program had to make several concerted efforts to help participating communities in developing their ability to prevent and control animal epidemics sustainably. These efforts included developing contingency plans for LGs to address abnormal animal deaths; establishing a revolving fund for a chicken vaccination campaign; and providing knowledge via a “Training on Prevention and Control of Disease in Chickens” program to chicken raisers about prevention and control of chicken diseases, including the method of vaccinating their animals in a proper and timely manner.

The Training on Prevention and Control of Disease in Chickens program was an integrated effort among LGs, the PODD project team, and district DLD offices. Knowledge of important chicken diseases, including avian influenza, Newcastle disease, infectious bronchitis, and infectious bursal disease, was provided to chicken farmers in the program. It started with meetings being conducted with all relevant stakeholders to develop an operational plan. Once the plan was developed, it was forwarded to all LGs and the director of the PODD project to request financial support. The LGs and the PODD project team provided equal financial support. After financial approval, communities conducted group meetings to specify the directions of operation for providing services such that the communities would play a role and then conducted activities according to the plan. At the end of the program, the program was evaluated with respect to the reduction of chicken deaths and sickness; the use of funds; and the participation of the community members, the PODD project team, and the LG. A total of 20 LGs have run this program in their community.

### Initial Focus on Priority Animal Diseases

Because animal infectious diseases are the primary focus of PODD surveillance, a specific set of priority animal diseases was incorporated into the PODD app initially (Textbox 1). Volunteer reporters were trained to detect either symptoms of these diseases or the diseases themselves (ie, based on case definitions, as presented in Textbox 1) and enter their observations into the PODD system. After 1 year, human diseases and environmental problems were added to the tool (see Textboxes 2 and 3). The list of animal diseases, human diseases, and environmental problems is provided in the PODD mobile app for early detection of problems, which have potential subsequent effect on another component in One Health.

**Animal diseases and case definitions used in the Participatory One Health Disease Detection (PODD) mobile app.**

- Poultry (chicken and duck)
  - Fowl cholera: sudden death and rapid spread
  - Fowl pox: pus or black pimple on the face; death possible
- Pig
  - Foot and mouth disease: blister or wound at hoof and mouth; salivation; pain
  - Brucellosis: abortion in many sows
  - Influenza: coughing; dyspnea; runny nose
  - Leptospirosis: jaundice, yellowish eyes; bloody urine (hematuria)
- Ruminants (cow, buffalo, goat, and sheep)
  - Anthrax: sudden death; bleeding from body opening
  - Foot and mouth disease: blister or wound at hoof and mouth; salivation; pain
  - Brucellosis: abortion in many animals
  - Leptospirosis: jaundice; yellowish eyes; bloody urine (hematuria)
  - Tuberculosis: emaciation; chronic coughing; pustules in carcass
- Dog and cat
  - Rabies: biting; aggression; inability to swallow; lack of coordination; seizure; death
  - Canine distemper: wet eyes; pustules in the belly; convulsion
  - Enteritis: vomiting; bloody diarrhea
- Horse
  - Influenza: coughing; dyspnea; runny nose
  - Surra: swollen legs; pale; cannot stand; bleeding in the eyes possible

**Human diseases, risks, and problems that volunteers can report via the Participatory One Health Disease Detection (PODD) mobile app.**

The following diseases are zoonoses with high impacts on human health:

- Infection caused by *Streptococcus suis*
- Leptospirosis
- Suspected bovine tuberculosis
- Influenza
- Trichinosis

The following are human diseases with high impacts on human health; they need to be controlled early to limit disease spreading:

- Hand, foot, and mouth disease
- Suspected dengue virus

The following are risks to food safety that can harm the quality of people’s lives. Reporters can take a photo, indicate location, and send the data to public health authority via the PODD app. The authorities must check and investigate all reports:

- Food poisoning
- Dirty restaurant/ butcher
- Toxic mushroom
- Suspected contaminated food (chemical/biological/physical)
- Low price meat
- Low quality food

The following problems, all of which still occur in several places across Thailand, were added to the PODD app for consumer protection and community monitoring:

- Unsafe/nonapproved cosmetics
- Fraudulent drugs
- Drugs containing steroids
- Unapproved drugs or herbs
- Reused cooking oil
- The sale of animal drugs without a sale certificate
- The sale of unapproved animal drugs

The following was added to the PODD app to monitor the risk of rabies in communities. Reporters report humans who have been bit by animals, particularly dogs and cats, and then follow the animals for 10 days after the biting. If the animal dies within 10 days, they immediately report the death to public health officers in their community:

- Animal bite

**Environmental problems that volunteers can report via the Participatory One Health Disease Detection (PODD) mobile app.**

The following pollution types are very common in many communities across Thailand, especially in the urban areas. Reported data are sent to local government (LGs) to check and solve the problems:

- Loudness
- Bad smell
- Smog
- Water pollution
- Garbage
- Mosquito reproductive source

The following natural disasters generally occur in rural or suburban areas. Because reported data are sent directly to LGs, LGs can use this part of the POD app for the monitoring and early detection of disasters in their communities:

- Outdoor burning
- Forest fire
- Flooding
- Landslide

## Results

### Performance Evaluation of Local Governments and Reporting Volunteers

After 12 months of PODD system implementation, outcome mapping was used to evaluate the changing behaviors, relationships, activities, and actions of the LGs and volunteers involved with the PODD system [5]. The LG and volunteer performances were classified using the results of the outcome mapping process. Criteria used to evaluate the level of achievement of LGs included LG enthusiasm, endorsement and recognition of the PODD system, and resources donated. Evaluation criteria for volunteer reporters included regularity of reporting, participation in training, leadership, enthusiasm, and possibility of being a role model for other volunteers.

On the basis of these evaluations, 3 groups of LGs (A-C) and 4 groups of volunteers (A-D) were identified. Among LGs, those categorized into the A group were very active with the PODD project. The B group LGs had achieved PODD project requirements or had met PODD project requests but were not as active. The group C LGs were least involved with the PODD project. The majority of LGs (34/74, 46%) were categorized into group B. Although they were willing to participate in the project and ran PODD activities in their community at the request of PODD, they were not proactive with respect to initiating the activities by themselves. The least number of LGs were in group C (15/74, 20%; Table 1).

Among volunteer reporters, group A reporters were those who showed excellent performance. Group D reporters showed the poorest performance. After 1 year of implementation, 20.0% (60/300) of volunteer reporters showed great performance with group A. Most reporters were categorized as either group B (104/300, 34.7%) or C (113/300, 37.7%; Table 2). These reporters were those who attended PODD training and activities and regularly reported abnormal events but were not as proactive as reporters in group A. Only about 7.7% (23/300) were categorized as group D. Group D performers could improve their performances if further training and learning sources were provided.

**Table 1:** The proportion of local governments in each group (A-C), with group A being the most engaged in Participatory One Health Disease Detection (PODD) and group C being the least engaged after 1 year of PODD implementation.

Group	Local government, n (%)
A	25 (34)
B	34 (46)
C	15 (20)

**Table 2:** The proportion of volunteer reporters in each group (A-D), with group A being the most engaged and group D being the least engaged after 1 year of Participatory One Health Disease Detection (PODD) implementation.

Group	Volunteer reporters, n (%)
A	60 (20.0)
B	104 (34.7)
C	113 (37.7)
D	23 (7.7)

### System Effectiveness in Early Detection and Rapid Response to Abnormal Deaths in Backyard Chickens

The primary goal of the PODD system is to detect emerging diseases in animals before it spreads to humans; thus, PODD primarily focused on animal population rather than human population. During the first 16 months of PODD operation, a total of 113,911 reports were sent by PODD volunteers. Of these, 98.82% (112,571/113,911) indicated that conditions were normal in their communities, whereas 1.18% (1340/113,911) reported abnormal events. Among the abnormal event reports, 76.79% (1029/1340) were confirmed to be true reports, whereas the remainder were found to be false positives or human error reports. The majority of abnormal reports were of sick or dead animals (404/1029, 39.26%), followed by zoonoses and other human diseases (129/1029, 12.54%), environmental problems (112/1029, 10.88%), and animal bites (110/1029, 10.69%). Among sick and dead animal reports, chicken (140/404, 34.6%) and cattle (132/404, 32.7%) were the two most frequently reported species.

Upon further investigation of these abnormal event reports, a total of 36 disease outbreaks, all in backyard animals, were detected by the PODD system during its first 16 months. These included 26 chicken disease outbreaks, 4 cattle disease outbreaks, 3 pig disease outbreaks, and 3 fish disease outbreaks.

### Case Study: Participatory One Health Disease Detection Controls a Backyard Chicken Disease Outbreak, March 2016

A chicken disease outbreak occurred in a 50-chicken household in Ping Kong, Chiang Dao District, in the northern part of Chiang Mai. In this household, 25 chickens were sickened and the other 25 died. The outbreak was reported through the PODD app by a volunteer in mid-March 2016. Clinical signs in the chickens included convulsion, paralysis, diarrhea, edema eyelids, depression, loss of appetite, and sudden death. The PODD volunteer reported similar clinical signs in 10 additional households near the index case household. The PODD epicenter verified the cases and classified them as a “suspected outbreak.” All local stakeholders, including provincial and district DLD officers, the LG mayor and staff, and PODD staff and researchers, were notified; a disease investigation team was sent into the field 3 days later. This response was faster than it would have been through a traditional (ie, nonparticipatory) surveillance process.

The disease investigation team determined that similar cases had occurred 2 weeks before in 20 additional households, all located within the same small area, and that each of those households had lost 5 to 10 chickens.

After the LG received notification from the PODD epicenter, the LG staff went to the affected area and disinfected it. The Chiang Dao District DLD officer and epicenter staff collected lab samples and provided vitamins and medicines to the chicken owners. No additional sick or dead chickens were reported 7 days after the first report was sent to the epicenter. Thus, the outbreak was controlled. Even though this case was animal disease, public health authorities were informed for monitoring similar clinical signs in humans.

However, the cause of the outbreak was not confirmed. Several essential chicken vaccines, including vaccines for Newcastle disease, infectious bronchitis, and infectious bursal disease, were administered to chickens in the community. Additionally, the Ping Kong LG collaborated with the Chiang Dao District DLD office to train chicken owners in the community about disease prevention, especially the vaccination program. Villagers and chicken owners, who were affected by the outbreak, responded to the LG staff, DLD officer, and PODD staff in a positive way.

## Discussion

Disease surveillance is important for the early detection of disease, particularly emerging diseases, in a population [6]. Both human and animal health authorities also use disease surveillance as a main tool for monitoring diseases. Since the outbreak of severe acute respiratory syndrome in 2003 and the emergence of highly pathogenic avian influenza (subtype H5N1) between 2003 and 2005, disease surveillance has played an important role in global health security.

Significant progress in communication technology has led to several new disease surveillance technologies that can complement the traditional reporting systems. The Program for Monitoring Emerging Diseases, a global list that serves in infectious disease reporting [7], and the Global Public Health Information Network, a news aggregation system that detects early signs of disease outbreaks [8], were two of the first efforts to leverage the Internet for global infectious disease surveillance. Other systems such as HealthMap, MedISys, and Biocaster have also been developed, using a variety of digital media and a blend of computer algorithms and human expertise to detect the first signs of disease transmission [9]. In addition to these more passive data collection approaches, active participatory disease surveillance has been evolving.

In this paper, we have described the initial achievements of PODD, a new disease surveillance tool that integrates One Health, community participation, and digital technology (ie, a mobile app) for the early detection and management of emerging zoonotic pathogens, since their inception, in backyard animal populations. In PODD, community members play an important role in disease surveillance. Due to the involvement of the LGs and villagers (eg, volunteer reporters) in the PODD project, communities are empowered to seek solutions and solve challenges by themselves [10]. On the basis of its 12-month outcome mapping and evaluation, the PODD research team was able to better understand motivations of both the LGs and volunteers and choose appropriate strategies to strengthen the capacities of the LGs and volunteer reporters to conduct disease surveillance in their communities. In March 2016, the collaborative use of PODD by the community and DLD officers enabled early detection of a backyard chicken disease outbreak, which led to an immediate response that reduced the size and spread of the outbreak. The outbreak was controlled within 7 days, which was faster than the time taken by a traditional system to respond.

PODD has also applied the One Health approach to the new disease surveillance system by integrating the collaboration among transdisciplinary stakeholders and linking the cooperation between animal, human, and environmental sectors. In Tanzania, the Epicollect mobile app was implemented, with the One Health approach, participatory epidemiology, and technology, for infectious disease surveillance. The study indicated that this approach is suitable for countries with limited resources and reduces the time of disease detection in both animals and humans [11]. Another study recommends that health policy decisions must be made in sync with community if One Health approach is applied for infectious disease surveillance [12]. PODD has begun in resource-limited areas, such as Thailand, and has engaged stakeholders from different levels, from community members to policy makers. These procedures could assist emerging disease control in communities where PODD is implemented.

The participatory surveillance with digital technology through community commitment showed several advantages, including timeliness, affordability, and scalability, particularly in resource-limited areas [13,14]. PODD system is another tool that provides these advantages to the community. PODD integrated the efforts of communities, authorities, and policy makers to prevent health problems through participation. It could be expanded to all area in Thailand to gain those advantages.

Although PODD has been researched and developed for 2 years, it needs to be further developed. In the future, the PODD project will work to reduce the time required to respond to the reports and to improve the specificity and sensitivity of disease detection, particularly for animal diseases. For example, as part of the effort to improve disease detection, reports from the PODD system could be compared with reports from official sources, such as animal disease reports from the Department of Livestock or human disease reports from the Ministry of Public Health. The existing system needs to be integrated with upcoming technology for sustainability.

## Abbreviations

AI: avian influenza
DLD: Department of Livestock Development
LG: local government
PODD: Participatory One Health Disease Detection
SMS: short message service
